# Cancer-related genes in the transcription signature of facioscapulohumeral dystrophy myoblasts and myotubes

**DOI:** 10.1111/jcmm.12182

**Published:** 2013-12-17

**Authors:** Petr Dmitriev, Ulykbek Kairov, Thomas Robert, Ana Barat, Vladimir Lazar, Gilles Carnac, Dalila Laoudj-Chenivesse, Yegor S Vassetzky

**Affiliations:** a UMR8126, Université Paris-Sud 11, CNRS, Institut de cancérologie Gustave RoussyVillejuif, France; b INSERM U1046, Université Montpellier IMontpellier, France; c Department of Genomic and Personalized Medicine, Center for Life Sciences, Nazarbayev UniversityAstana, Kazakhstan; d National Scientific Shared Lab of Biotechnology, National Center for BiotechnologyAstana, Kazakhstan

**Keywords:** cancer, rhabdomyosarcoma, Ewing's sarcoma, FSHD, muscular dystrophy

## Abstract

Muscular dystrophy is a condition potentially predisposing for cancer; however, currently, only Myotonic dystrophy patients are known to have a higher risk of cancer. Here, we have searched for a link between facioscapulohumeral dystrophy (FSHD) and cancer by comparing published transcriptome signatures of FSHD and various malignant tumours and have found a significant enrichment of cancer-related genes among the genes differentially expressed in FSHD. The analysis has shown that gene expression profiles of FSHD myoblasts and myotubes resemble that of Ewing's sarcoma more than that of other cancer types tested. This is the first study demonstrating a similarity between FSHD and cancer cell expression profiles, a finding that might indicate the existence of a common step in the pathogenesis of these two diseases.

## Introduction

Recent studies have shown that murine models of Duchenne muscular dystrophy (DMD) and Limb-girdle muscular dystrophy (LGMD) frequently develop cancer. Mice with mutations in the Dystrophin (DMD), Calpain-3 (LGMD2A) or Dysferlin (LGMD2B) genes are susceptible to malignant tumours originating from the skeletal muscles 1–4 (reviewed in 5).

Several cases of coincidence of cancer and DMD 6–13 or facioscapulohumeral dystrophy (FSHD) 14–16 have been also reported in humans; however, at the moment, DMD and FSHD patients are not considered to be more susceptible to cancer than the general population. Myotonic dystrophy (MD) patients are known to have higher risk of cancer 17,18, while no cancer cases are known to be reported among LGMD patients.

Here, we focused on FSHD, an autosomal dominant hereditary neuromuscular disorder, because it is genetically associated with the same genomic region that is re-arranged or epigenetically modified in various types of cancer. The majority of FSHD patients carry a deletion of the 3.3 kb-long D4Z4 macrosatellite repeats, accompanied by DNA demethylation and chromatin structure alterations within the subtelomeric region of chromosome 4 (4q35) 19,20 (for review see 21). The minor form of FSHD (FSHD2) is not associated with D4Z4 repeat contractions, but shares common epigenetic alterations in 4q35 with the major form (FSHD1) of this disease 22.

The rearrangement of the FSHD-associated region in 4q35 has been also found in various tumours, including undifferentiated soft tissue sarcoma 23–25, Ewing's sarcoma 26 and rhabdomyosarcoma 27. Epigenetic alterations of the same region have been documented in cervical and ovarian cancers 28,29.

D4Z4 repeats encode a powerful transcription regulator, double homeobox protein 4 (DUX4) 30, playing an important if not the key role in the aetiology of FSHD 31 and a potent enhancer that is capable of regulating a variety of genes 32,33. Interestingly, the expression level of DUX4 is altered in cervical cancer 28 and in Ewing's sarcoma where this gene functions as a chimeric oncogene if fused to CIC gene as a result of the t(4;19)(q35;q13.1) translocation 26.

The involvement of 4q35 in FSHD and several types of cancer prompted us to search for similarities in gene expression profiles of FSHD and cancer cells and tissues. We have found that a significant number of genes differentially expressed in cancer are also differentially expressed in FSHD and, according to statistical criteria, this phenomenon could not be explained by a simple coincidence. We then searched for similarities of expression profiles of FSHD and various types of cancer and found the highest resemblance between FSHD and Ewing's sarcoma expression profiles.

Taken together, the results of this study establish for the first time a link between FSHD and cancer at the level of gene expression.

## Materials and methods

### Cell culture

Primary human myoblasts from normal individuals and FSHD patients (Table S6) Primary human myoblasts were isolated from skeletal muscles of healthy individuals as described in 34, for details see Table S4), purified with an immuno-magnetic sorting system (MiltenyiBiotec, Bergisch Gladbach, Germany) by using an anti-CD56/NCAM antibody according to the manufacturer's specifications. CD56-positive myoblasts were seeded in collagen-coated Petri dishes (P1) and cultured in DMEM, 10% FCS, 1% Ultroser G, at 37°C with 5% CO_2_. All experiments were carried out between P1 and P5 to avoid cell senescence. Myoblast purity was determined by staining for Desmin.

### Gene expression analysis

Total RNA was isolated from 2 × 10^6^ myoblasts or myotubes by using Trizol (Invitrogen, Carlsbad, CA, USA), 400 ng of total RNA was reverse transcribed by using the High Capacity cDNA Archive kit (Applied Biosystems, AB, Foster city, CA, USA) according to the manufacturer protocol. cDNA was mixed with 2× TaqMan PCR mix (AB) and amplified with TLDA (Taqman Low Density Array; AB) by using Abiprism 7900HT. The expression was analysed by using the ΔΔCt method 35.

### Statistical analysis

qRT-PCR data have been analysed by using one-way anova 36, *P* < 0.05 have been considered significant. To calculate the significance of the lists’ intersection, we have used the LOLA tool (http://www.lola.gwu.edu). For the intersection of two lists, the *P*-value is determined as the probability of observing an intersection size that occurs by chance and is larger than the given one 37.

### Lists of genes

The statistical significance of an intersection of gene lists has been calculated using online service List Of Lists Annotated (LOLA) http://www.lola.gwu.edu/ 37. To standardize the lists of genes extracted from different publications, we have used GenBank GeneIDs as reference points. As the GeneID was not usually provided along with the expression data, we have converted available Affymetrix or Ensembl IDs or Gene names to GeneID by using db2db online tool http://biodbnet.abcc.ncifcrf.gov/db/db2db.php 38. In the cases where Gene name was not recognized by the db2db service, we assumed that the authors of the study used a synonym. In these cases, we provide both Gene names: the major one, recognized by db2db service and the synonym from the original publication.

#### FSHD-related genes

List A was created by using supplementary data from 39, previously available at http://www.ucihs.uci.edu/biochem/winokur under ‘publications’ (Table S2). To our knowledge, at the moment of submission, the data are no longer available for downloading on this server. Genes with fold change >2 and *P* < 0.05 were considered as differentially expressed.

In total, 527 probes from the two types of microarrays used in the study (HuFL and U95) corresponding to differentially expressed genes were retained by the authors. To standardize this list and make it compatible with the lists originating from other studies, we have transformed probe codes to GeneIDs. Some of probe codes corresponded to multiple genes and some probe codes could not be found in available databases, therefore, the resulting list counted 529 unique gene IDs, 301 up-and 238 down-regulated. List B was created by using supplementary data from 40 previously available at http://www.ucihs.uci.edu/biochem/winokur under ‘publications’. To our knowledge, at the moment of submission, the data are no longer available for downloading on this server. Using Affymetrix GCS V3.2 software and undisclosed statistical criteria, the authors attributed an arbitrary value of 1.1 to transcripts exhibiting an increase (I), 1.0 – marginal increase (MI), 0.0 – no change (NC), −1.0 – marginal decrease (MD) and −1.1 – decrease (D). We summed these values across all pairwise comparisons and considered transcripts with a sum of >5.5 as up-regulated and transcripts with a sum of <−5.5 as down-regulated in FSHD. In total, 236 probes codes corresponding to differentially expressed genes were selected according to these criteria. After transforming the list of probe codes to a list of GeneIDs, we obtained a list of 297 unique entries (26 up-and 271 down-regulated GeneIDs). Lists Ca, Cb and Cc were created by using supplementary tables A–G from 41 available for downloading at http://gefu.cribi.unipd.it/papers/FSHD/. From these tables, we have retrieved symbols of genes differentially expressed in FSHD biopsies divided into three sublists of genes (a, b and c), corresponding to the groups of patients, containing 194, 164 and 164 gene names respectively. These were converted to three lists of GeneIDs with 207 (112 up-and 95 down-regulated), 177 (118 up-and 59 down-regulated) and 177 (106 up-and 71 down-regulated) unique GeneIDs respectively. List D was created by using data from supplementary table from 42 available at http://onlinelibrary.wiley.com/doi/10.1634/stemcells.2007-0465/suppinfo. The probe names corresponding to the genes differentially expressed in FSHD mesoangioblasts were converted to 32 unique GeneIDs (20 up-and 12 down-regulated). Lists Ea and Eb were created from Tables S3 and S4 from 43 available at http://www.neurology.org. The probes corresponding to differentially expressed genes were further subdivided into two sublists a and b, corresponding to the genes differentially expressed in FSHD and other types of muscular dystrophy (318 probes) and genes differentially expressed in FSHD only (134 probes). After the transformation of probe names to GeneIDs, these lists were converted to the Lists Ea and Eb containing 376 and 176 unique GenesIDs respectively. Lists Fa and Fb were created from Table S1 in 44 available for downloading at http://www.plosone.org/article/info+3Adoi+2F10.1371+2Fjournal.pone.0020966. Lists of Ensembl IDs of genes differentially expressed in myoblasts or myotubes from FSHD1 patients have been transformed to the two lists of unique GeneIDs containing 393 and 111 unique entries respectively. Lists Ga and Gb were extracted from Table S1 from 45 available online at http://www.pnas.org/lookup/suppl/doi:10.1073/pnas.1209508109/-/DCSupplemental/sd01.xls. The authors have analysed transcriptome profiles of biceps, muscles affected in FSHD and deltoids that are usually spared in FSHD patients and compared them to transcriptome profiles of the same types of muscles from normal subjects. The authors have found 238 GeneIDs differentially expressed (1.2-fold change, *P* < 0.01) in affected FSHD biceps, but not deltoids as compared with normal biceps and 182 GeneIDs differentially expressed according to the same statistical criteria in both biceps and deltoids of FSHD patients as compared with normal subjects. We have extracted these lists from a single Table S1 and named them List Ga and List Gb respectively.

#### Cancer-related genes

List 1 was constructed by using the data from 46 (Table S1). The authors of the study have conducted a meta-analysis of transcriptomes of 2186 samples representing 20 different cancers from 39 studies and obtained a transcriptome signature comprising 187 genes (list 1 of the present study) of which 117 up-regulated (Table 1 in 46) and 70 were down-regulated (Table 2 in 46). Authors claimed that this transcriptome signature could discriminate cancer samples with 92.6% accuracy independently from their tissue of origin. List 2 was constructed by using the data from Table 1 in 47. The authors of the study used a published census of cancer-related genes 48 to select a representative list of 56 cancer-related genes. List 3 have been created by using data from 49. The authors of the study have performed transcriptome profiling of 373 samples of 15 different types of benign and malignant tumours by using 17.5K custom-made cDNA arrays. These arrays allowed to cover the expression of 12,947 genes that included 332 cancer biomarkers (Table S1 in 49) known from literature. The expression analysis of these 332 cancer biomarkers allowed the authors to select from it 56 genes (49 does not precise which genes these were) that could discriminate between benign and malignant tumours with 88% accuracy. List 4 was created by using data from 50. The authors have conducted a meta-analysis of transcriptomes of 3209 samples that contained normal tissues, immortalized cell lines, a variety of cancers pluripotent and partially committed stem cells. As a result, the authors obtained a list of 189 genes (stem cell gene set, Tables S1–S4 in 50) that could be used as a quantitative measure of stem cell-associated transcriptional activity and could also discriminate histological grades for a variety of human malignancies 50. List 5 has been created by using the data from 51. The authors performed transcriptome profiling of five cell lines representing altogether two different systems of cell transformation, one based on an inducible expression of v-Src and another one based on inducible expression of small and large T antigens of SV40. Common transcriptional profile of these cell lines consisted of 343 genes (238 up-and 105 down-regulated, Table S3 in 51). List 6 is based on the data from 52. The authors conducted a meta-analysis of 36 transcriptome signatures of tumours originating from 12 representative tissue types. This analysis resulted in identification of a common transcriptome signature of 183 genes from which 67 most significant genes (Fig. 2 in 52) were differentially expressed in nearly all cancer types available to the authors. List 7 was created from our own large-scale literature search.

**Table 1:** The lists of cancer-related genes used in the current study. For full lists of genes refer to Table S1

List	Reference	Samples	Cancers types	Original gene list	Unique GeneIDs	Platform
1	46	2186	20	187 genes (117 genes up-and 70 down-regulated in cancer)	190	Meta-analysis
2	47	N/A	N/A	56 genes (22 oncogenes and 34 tumour suppressors) causally related to cancer	56	Literature search
3	49	N/A	N/A	332 genes	277	Literature search
4	50	3209	Unknown	189 genes	189	Meta-analysis
5	51	5	2 model systems of cell transformation	343 genes (239 up-and 104 down-regulated in cancer)	348	Affymetrix U133 2.0A
6	52	3700	12	183 genes (67 most significant)	80	Meta-analysis
7	This study			400 genes		Literature search

**Table 2:** Lists of genes differentially expressed in FSHD. For full lists of genes refer to Table S2

List	Reference	Samples	Patients analysed					Deregulated genes (probes)	Recognized GeneIDs	Statistical criteria	Microarray
A	39	Biopsies	Three FSHD patients, three normal participants, pairwise comparisons					230	Total: 529; Up: 301; Down: 238	Twofold change, *P* < 0.05	Affymetrix HuFL GeneChip
			Nine FSHD patients, six normal participants, pairwise comparisons					297			Affymetrix U95A GeneChip
B	40	Myoblasts	Four FSHD patients, three normal participants, pairwise comparisons					236	Total: 296 Up:26; Down: 270	Not described	Affymetrix Hu6800 GeneChip
Ca	41	Biopsies	11 FSHD patients	Group A (>4 D4Z4 repeats)				194	Total: 207; Up: 112; Down: 95	SD<0.5 for single patient gene replicate	Custom cDNA array
Cb				Group B (3-4 D4Z4 repeats)				164	Total: 177; Up: 118; Down: 59		
Cc				Group C (1 D4Z4 repeat)				164	Total: 177; Up: 106; Down: 71		
D	42	Mesoangioblasts	Two FSHD patients, two normal participants					32	Total: 32; Up: 20; Down: 12	Not described	HG-Focus GeneChip Affymetrix
Ea	43	Biopsies	19 FSHD, 11 MD patients and 30 normal participants	FSHD and MD *versus* Normal				318	Total: 326; Up: 186; Down: 140	1.5-fold change, *P* < 0.001	Affymetrix HG133A HG133B
Eb				FSHD, not MD *versus* Normal				134	Total: 156; Up: 51; Down: 105		
Fa	44	Myoblasts	Seven FSHD1 patients, two FSHD2 patients, eight normal participants	FSHD1 *versus* control				367	Total: 395; Up: 133; Down: 262	Twofold change; *P* < 0.01	Affymetrix human exon 1.0 ST arrays
Fb		Myotubes		FSHD1 *versus* control				129	Total: 111; Up: 61; Down: 50		
Ga	45	Biopsies	29 FSHD patients, 21 normal participants	FSHD biceps AND deltoid				272	Total: 272; Up: 141; Down: 131	*P* < 0.01, 1.2-fold change	Affymetrix GeneChip Gene 1.0 ST arrays
Gb				FSHD biceps NOT deltoid				162	Total: 162; Up: 58; Down: 104		

Expression profile scoring of FSHD samples has been conducted as described in 53 for expression profiles of tumour tissue samples. In total, 96 genes have been used for scoring, for each of them, the authors have assigned a rank R, different for each of the four categories, EWS, RMS, NB and BL (Table S3 in 53 and Table S5 in the present study). The contribution of a given gene to scoring is inversely proportional to its rank. To contribute to a given category, the level of gene expression must correspond to the sign in the scoring table *e.g*. ‘+1’ corresponds to up-and ‘−1’ to down-regulated genes. To determine the contribution of a gene to a given category, the following schema was used. If the level of expression of a gene corresponds to the sign in a given category in the scoring table, the contribution B of the gene to a given tumour category is calculated according to formula B = 1 – C × R where C is the coefficient and R is the rank. The coefficient was calculated independently for every category as follows: C = 1/HR where HS is the highest rank for a given category resulting in C_EWS_ and C_not EWS_ = 0.000543, C_RMS_ and C_not RMS_ = 0.000499, C_NB_ and C_not NB_ = 0.000445, C_BL_ and C_not BL_ = 0.000980. If the level of expression of a given gene did not correspond to the sign in the scoring table, the contribution of a gene to a given category was considered 0. To determine the score of an expression profile, scores of every gene are summed. Using these formulas, we have calculated the scores of ‘ideal’ tumours (where all genes have the direction of expression corresponding to the signs in the scoring table). The resulting scores are: S_EWS_ and S_not EWS_ = 62.644; S_RMS_ and S_not RMS_ = 64.041; S_NB_ and S_not NB_ = 70.408; S_BL_ and S_not BL_ = 70.243.

## Results

### Lists of cancer-and FSHD-related genes

We have first compiled seven lists containing 56–343 published cancer-related genes (Table 1). These lists resulted either from a meta-analysis of transcriptomic data (Lists 1, 4 and 6), direct transcriptome analysis of cancer samples (List 5) or were assembled from cancer-related genes known from the literature (Lists 2 and 3). List 7 has been specifically created for this study by using bibliographical search of genes that have been previously used as biomarkers in various types of cancer. In total, Lists 1–7 assimilate transcriptome profiles of 9100 samples representing 35 different cancer types (Table S1).

Twelve lists of genes differentially expressed in FSHD have been extracted from six previously published transcriptome studies and one combined transcriptome–proteome 41 study (Table 2). In total, these lists represent transcriptional profiles of 86 FSHD patients and 73 healthy controls. Of these, eight lists of differentially expressed genes resulted from transcriptome analysis of skeletal muscle biopsies of FSHD patients (Lists A, Ca, Cb, Cc, Ea, Eb, Ga, and Gb), two lists resulted from the analysis of FSHD primary myoblasts (List B, Fa), one list resulted from the transcriptome analysis of FSHD myotubes (List Fb) and one list resulted from the transcriptome analysis of mesoangioblasts isolated from FSHD patients (List D; Table S2).

### Cancer-related genes are differentially expressed in FSHD

Based on the fact that the same genomic region is involved in the pathogenesis of both FSHD and cancer, we hypothesized that FSHD and cancer expression profiles might have a significant number of the common differentially expressed genes.

To test our hypothesis, we have systematically searched for the common genes shared by the lists of genes differentially expressed in FSHD and cancer-related, and found a statistically significant overlap between these lists (Table 3). Interestingly, the significance of these intersections depended on the type of samples used for transcriptome profiling and the list source. The lists that were produced from meta-analysis of cancer samples (*e.g*. Lists 1, 4 and 6) have demonstrated the most significant overlap with List Fa and List B, both of which are composed of genes differentially expressed in FSHD myoblasts (Table 3). Lists that were produced from simple literature search of cancer-related genes, *e.g*. the Lists 2, 3 and 7, gave the highest statistically significant intersection with the List A composed of genes differentially expressed in FSHD biopsies.

**Table 3:** *P*-values of intersection of gene lists of FSHD-and cancer-related genes. For the expanded version of this Table refer to Table S3

		List 1	List 2	List 3	List 4	List 5	List 6	List 7
	Total genes/list	190	56	277	189	348	80	576
List A	539	6 (ns)	7***	30***	11***	7***	4*	49***
List B	297	2**	3**	15***	3***	2***	1***	20***
List Ca	207	1 (ns)		15***		1 (ns)		10**
List Cb	117			14***		2*		9**
List Cc	177			14***		2*		9**
List D	32	1 (ns)			1 (ns)	*		3*
List Ea	376	7*	1 (ns)	15***	9*	9***		15***
List Eb	176	1 (ns)	1 (ns)	5**	3 (ns)	4**		8**
List Fa	395	14***	3*	12***	24***	5***	9***	23***
List Fb	111	2 (ns)		4*	2 (ns)	1**	1 (ns)	4 (ns)
List Ga	272	2 (ns)		13***	1 (ns)	1 (ns)		11**
List Gb	220	2 (ns)		8***	1 (ns)	1 (ns)		9**

* P*<*0.05;

** P*<*0.01;

*** P*<*0.001.

The number of cancer-related genes among genes differentially expressed in FSHD varied for different studies. For some (*e.g*. Lists A, B and Fa), a highly significant intersection with all the cancer-related lists has been observed. For others, we could obtain a statistically significant intersection with only a few lists of cancer-related genes (Table 3). The Reasons for this discrepancy are unknown and probably linked to the study design. The description of cancer-related genes that have been found among genes differentially expressed in FSHD can be found in the Table S4.

Finally, we have observed a much higher intersection of the lists of cancer-related genes with the list of genes differentially expressed both in FSHD and MD (List Ea) as compared with the other lists from the same study (*e.g*. List Eb), that contained genes differentially expressed exclusively in FSHD. The latter result confirms the known link of MD with cancer and indicates that MD and FSHD share the same cancer-related genes (Table 3).

Next, we tested whether cancer-related genes were up-or down-regulated in FSHD as compared with the control. This analysis did not produce a clear result as we could find examples of the presence of cancer-related genes within lists of genes both up-and down-regulated in FSHD (Table S3).

### Similarity between FSHD and Ewing's sarcoma gene expression profiles

**Fig 1:**
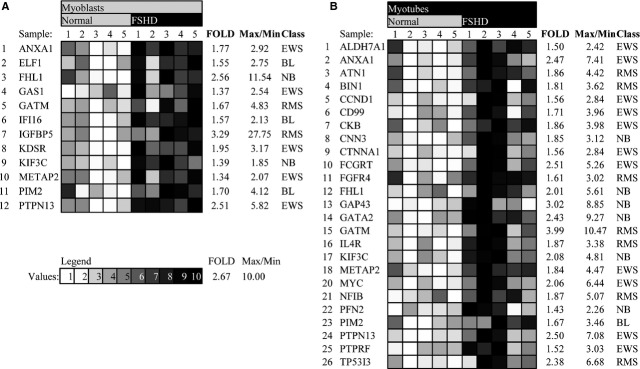
Cancer-classifier genes differentially expressed in facioscapulohumeral dystrophy (FSHD) myoblasts (A) and myotubes (B). Genes that are differentially expressed both in FSHD myoblasts and myotubes as compared to healthy controls are shown in bold. (C) grey intensity corresponds to the expression level of the genes with darker squares corresponding to higher expression levels. The Sample description can be found in Table S6.

In addition, we tried to find out whether FSHD transcription signature resembles that of a specific cancer. We have selected 96 genes that were previously shown to be sufficient for classification of four types of cancers in humans: Ewing's sarcoma, rhabdomyosarcoma, neuroblastoma and Burkitt's lymphoma 53 (for review see 54) (Table S5) and tested their expression in myoblasts and myotubes from FSHD patients. We have found that 12 of these genes are up-regulated in FSHD myoblasts and 26 are up-regulated in FSHD myotubes as compared with the control. Of these, five genes including ANXA1, GATM, METAP2, PIM2 and PTPN13 are up-regulated both in FSHD myoblasts and in differentiated FSHD myotubes, seven genes including ELF1, FHL1, GAS1, IFI16, IGFBP5, KDSR and KIF3C were specifically up-regulated in FSHD myoblasts, while 20 genes (ALDH7A1, ATN1, BIN1, CCND1, CD99, CKB, CNN, CTNN1, FCGRT, FGFR4, FHL1, GAP43, GATA2, IL4R, KIF3C, MYC, NFIB, PFN2, PTPRF, TP53I3) were specifically up-regulated in FSHD myotubes (Fig. 1).

Interestingly, five of 12 cancer-classifier genes differentially expressed in FSHD myoblasts were specific to Ewing's sarcoma (EWS), while only 2/12, 2/12 and 3/12 of cancer classifiers were specific to rhabdomyosarcoma, neuroblastoma and Burkitt's lymphoma respectively (Fig. 1). In FSHD myotubes, 11 of 26 differentially expressed genes were specific to Ewing's sarcoma, seven of 26 were specific to rhabdomyosarcoma, six of 26 were specific to neuroblastoma and only one of 26 were specific to Burkitt's lymphoma.

**Fig 2:**
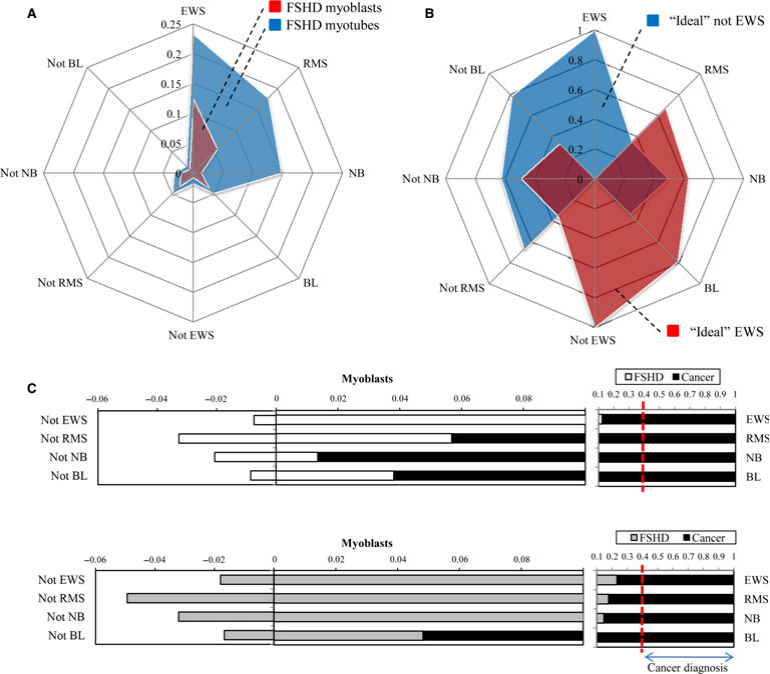
A radar chart indicating the similarity of the facioscapulohumeral dystrophy (FSHD) samples to the one of four frequent types of cancer (A). The numbers correspond to relative scores of FSHD gene expression profiles normalized to maximal scores of the ‘ideal’ expression signatures of tumour samples. (B) The same scoring system was applied to the ‘ideal EWS’ and the ‘ideal not EWS’ expression profiles. (C) Linear representation of the same data as in A. The limit, below which the algorithm cannot recognize the sample as a ‘true’ cancer is indicated to the right.

To quantitatively evaluate the similarity of the resulting gene expression profile of FSHD cells to cancer cell lines, we have used the scoring procedure described in 53. This procedure attributes a maximal score of 1 to each of the four evaluated cancers, and 0 to samples that are unrelated to cancer, scores between 0.4 and 1.0 being sufficient to establish a correct diagnosis 53 (Fig. 2B and C). The procedure attributed to FSHD myoblasts score 0.124 and to FSHD myotubes 0.233 for Ewing's sarcoma followed by rhabdomyosarcoma, neuroblastoma and Burkitt's lymphoma (Fig. 2A). This score was insufficient to result in a diagnosis of cancer (Fig. 2C); however, it clearly indicated that FSHD and cancer samples have similarities in their gene expression profile. Interestingly, transcription signatures of FSHD myoblasts and myotubes resembled those of Ewing's sarcoma more than other types of cancer (Fig. 2A).

## Discussion

A link between cancer and muscular dystrophy has been previously demonstrated in the murine models of Duchenne and Limb-girdle muscular dystrophies. Specifically, it was found that the mice with the mutation in Dystrophin, Dysferlin and Calpain-3 were susceptible to spontaneous formation of rhabdomyo-, fibro-and liposarcomas derived from skeletal muscle tissue 1–4 (reviewed in 5).

In human patients, only MD is currently associated with an increased risk of thyroid cancer and choroidal melanoma 17,18. Higher cancer risk in DMD patients was suspected, but not confirmed 2,13 To our knowledge, no such study has been conducted in the case of FSHD, the third most common muscular dystrophy in the world.

In the present work, we used meta-analysis to establish a link between FSHD and cancer at the level of gene expression. We have demonstrated that genes differentially expressed in FSHD contain more cancer-related genes than could be expected by chance. To exclude the possibility that these genes merely represent the tissue-specific markers unrelated to oncogenic process, we have selected studies that have analysed transcriptome signatures of a large number of cancer samples representing multiple cancer types of different origin.

We have also measured the expression level of 96 tumour markers and used a previously published scoring algorithm 53 to find out whether FSHD gene expression signature profile resembles any specific type of cancer. We expected, that the expression profiles of myogenic cells from FSHD patients would be more similar to the expression profile of rhabdomyosarcoma, a tumour originating from skeletal muscle tissue. Instead, we have found FSHD cells are more similar to Ewing's sarcoma. The resulting score, however, was insufficient to classify FSHD samples as true cancer samples, which is in agreement with the fact that none of the patients who participated in the study was diagnosed with cancer.

The reasons for the similarity of FSHD and tumour cell expression profiles remain unknown. One possibility is that this similarity originates from inflammation, fibrosis or oxidative stress that frequently accompany tumour development and are also observed in FSHD patients.

Alternatively, a certain similarity of FSHD and cancer gene expression profiles might be caused by the nature of the genetic defect in FSHD patients that affects the genomic region that is also altered in several types of cancer. The involvement of the same genomic region in both dystrophy and cancer is not unique to FSHD; this is also the case for the rearrangements in Dystrophin gene 55. Intriguingly, DUX4, a powerful transcription factor encoded in 4q35, is involved in the pathological mechanism of both FSHD 56 and Ewing's sarcoma 26.

Another plausible explanation of the similarity of FSHD and cancer gene expression profiles is based on pre-mRNA splicing. Pre-mRNA splicing is perturbed in FSHD 57,58 59, probably, because of elevated expression of FRG1, a novel splicing regulator 57,60. As alteration in pre-mRNA splicing is a common phenomenon in cancer 61,62, one could hypothesize that an abnormal pre-mRNA splicing might be a mechanism linking FSHD to cancer. In support of this hypothesis, we have observed that many cancer-related genes differentially expressed in FSHD are also differentially expressed in MD, a disease that has been shown to increase the risk of cancer in human patients 17,18. Myotonic dystrophy is caused by CTG and CCTG microsatellite repeat expansion affecting the function of splicing factors in the cells of the MD patients 63. Therefore, an altered pre-mRNA splicing could potentially initiate the process of tumour formation in MD; however, currently, this hypothesis is missing a solid experiment support 64,65.

The link at the level of gene expression that we have established between FSHD and cancer does not imply that FSHD patients might have a higher incidence of cancer, as compared to general population. Although several cases of cancer in FSHD patients have been reported, FSHD is not considered as a cancer-predisposing condition. The latter is supported by the mouse models of FSHD 57,66,67 that have not been reported to have higher than average incidence of tumours. However, the conclusions about cancer incidence in muscular dystrophy patients made using mouse models should be handled with caution. Indeed, high incidence of cancer observed in *mdx* mouse model is not supported by observations in human patients, who do not demonstrate higher incidence of cancer 2. Conversely, while the patients with MD suffer from an increased incidence of cancer, mouse models of this disease are not susceptible for cancer 68. To our knowledge, no cases of concomitant LGMD and cancer have been described, while LGMD mouse models are susceptible to cancer.

This discrepancy may be explained by certain limitations of mouse models to represent human diseases. For example, *mdx* mice do not suffer from muscle wasting and satellite cell pool depletion as human patients do. Therefore, high incidence of tumours in *mdx* mice might be explained by the regenerative environment of a permanently damaged muscle, favouring oncogenic transformation of muscle satellite cells in *mdx* mice, but not in DMD patients, where satellite cells are rare 2.

To conclude, we consider that to determine whether FSHD patients are more prone to cancer than general population, one should not rely on mouse models of this disease, but rather carry out a retrospective examination of medical histories of FSHD patients.

Our study may also have an impact on the development of FSHD therapy strategies. Recently, several anti-cancer drugs have been proven efficient in the mouse model of DMD 69,70. The similarity of gene expression profile linking FSHD to cancer, discovered in our study, may provide the basis for examination of the usability of anti-cancer agents in FSHD.

## Supporting information

Table S1 Lists of cancer-associated genes.

Table S2 Lists of genes differentially expressed in FSHD.

Table S3 Intersection of lists of cancer-related genes and lists of genes differentially expressed in FSHD.

Table S4 Description of cancer-related genes found among genes differentially expressed in FSHD.

Table S5 Scoring table for tumour classification.
