# Supplementary material for: Cancer-related genes in the transcription signature of facioscapulohumeral dystrophy myoblasts and myotubes
Source: J Cell Mol Med. 2013 Dec 17;18(2):208–17. doi: 10.1111/jcmm.12182 (PMC3930408; doi:10.1111/jcmm.12182)
Supplement: Table S4 — Description of cancer-related genes found among genes differentially expressed in FSHD. [file jcmm0018-0208-sd4.pdf]

List A			Cancer-related genes:							
			List 1	List 2	List 3	List 4	List 5	List 6	List 7	
GeneID	Gene name	FSHD	6	7	29	11	7	4	49	Gene description
70	ACTC1	UP			X					actin, alpha, cardiac muscle 1
8038	ADAM12	UP	X							ADAM metallopeptidase domain 12
125	ADH1B	DOWN			X					alcohol dehydrogenase 1B (class I), beta polypeptide
126	ADH1C	DOWN			X					alcohol dehydrogenase 1C (class I), gamma polypeptide
8165	AKAP1	UP			X					A kinase (PRKA) anchor protein 1
8644	AKR1C3	DOWN							X	aldo-keto reductase family 1, member C3
246	ALOX15	UP							X	arachidonate 15-lipoxygenase
301	ANXA1	UP			X					annexin A1
347	APOD	DOWN			X					apolipoprotein D
23365	ARHGEF12	UP							X	Rho guanine nucleotide exchange factor (GEF) 12
9823	ARMCX2	DOWN				X			X	armadillo repeat containing, X-linked 2
797	CALCB	UP			X					calcitonin-related polypeptide beta
801	CALM1	DOWN			X					calmodulin 1 (phosphorylase kinase, delta)
805	CALM2	DOWN			X					calmodulin 2 (phosphorylase kinase, delta)
23261	CAMTA1	DOWN			X					calmodulin binding transcription activator 1
64151	CAPG	UP					X			capping protein (actin filament), gelsolin-like
834	CASP1	UP							X	caspase 1, apoptosis-related cysteine peptidase
867	CBL	DOWN							X	Cbl proto-oncogene, E3 ubiquitin protein ligase
868	CBLB	UP							X	Cbl proto-oncogene, E3 ubiquitin protein ligase B
891	CCNB1	DOWN				X				cyclin B1
595	CCND1	UP			X				X	cyclin D1
1230	CCR1	UP				X				chemokine (C-C motif) receptor 1
960	CD44	UP			X					CD44 molecule (Indian blood group)
1050	CEBPA	DOWN							X	CCAAT/enhancer binding protein (C/EBP), alpha
3075	CFH	UP			X					complement factor H
1120	CHKB	UP			X					choline kinase beta
1152	CKB	UP			X					creatine kinase, brain
7123	CLEC3B	UP	X							C-type lectin domain family 3, member B
1277	COL1A1	UP							X	collagen, type I, alpha 1
1278	COL1A2	UP						X	X	collagen, type I, alpha 2
1397	CRIP2	UP						X		cysteine-rich protein 2
1436	CSF1R	DOWN							X	colony stimulating factor 1 receptor
1508	CTSB	UP							X	cathepsin B
1512	CTSH	UP				X			X	cathepsin H
1756	DMD	DOWN	X				X			dystrophin
2072	ERCC4	UP							X	excision repair cross-complementing rodent repair deficiency, complementation group 4
10160	FARP1	UP	X							FERM, RhoGEF (ARHGEF) and pleckstrin domain protein 1 (chondrocyte-derived)
2213	FCGR2B	UP							X	Fc fragment of IgG, low affinity IIb, receptor (CD32)
2329	FMO4	DOWN	X							flavin containing monooxygenase 4
2353	FOS	DOWN			X					FBJ murine osteosarcoma viral oncogene homolog
10468	FST	UP				X				follistatin
8522	GAS7	UP							X	growth arrest-specific 7
2719	GPC3	UP							X	glypican 3
3569	HGF	DOWN							X	hepatocyte growth factor (hepapoietin A; scatter factor)
8364	HIST1H4C	UP				X				histone cluster 1, H4c
3205	HOXA9	UP		X					X	homeobox A9
3492	IGH@	UP							X	immunoglobulin heavy locus
3493	IGHA1	UP							X	immunoglobulin heavy constant alpha 1
3494	IGHA2	UP							X	immunoglobulin heavy constant alpha 2 (A2m marker)
3500	IGHG1	UP							X	immunoglobulin heavy constant gamma 1 (G1m marker)
3501	IGHG2	UP							X	immunoglobulin heavy constant gamma 2 (G2m marker)
3503	IGHG4	UP							X	immunoglobulin heavy constant gamma 4 (G4m marker)
3587	IL10RA	UP				X				interleukin 10 receptor, alpha
3690	ITGB3	DOWN			X					integrin, beta 3 (platelet glycoprotein IIIa, antigen CD61)
182	JAG1	UP							X	jagged 1
3725	JUN	DOWN			X				X	jun proto-oncogene
3726	JUNB	DOWN				X			X	jun B proto-oncogene
3945	LDHB	UP			X					lactate dehydrogenase B
3936	LPL	UP							X	lipoprotein lipase
4212	MEIS2	DOWN			X					Meis homeobox 2
4291	MLF1	UP							X	myeloid leukemia factor 1
10962	MLLT11	DOWN					X		X	myeloid/lymphoid or mixed-lineage leukemia (trithorax homolog, Drosophila); translocated to, 11
4318	MMP9	UP			X			X	X	matrix metallopeptidase 9 (gelatinase B, 92kDa gelatinase, 92kDa type IV collagenase)
4609	MYC	DOWN		X	X				X	v-myc myelocytomatosis viral oncogene homolog (avian)
4763	NF1	UP		X					X	neurofibromin 1
4791	NFKB2	DOWN						X	X	nuclear factor of kappa light polypeptide gene enhancer in B-cells 2 (p49/p100)
3164	NR4A1	DOWN			X					nuclear receptor subfamily 4, group A, member 1
8013	NR4A3	UP							X	nuclear receptor subfamily 4, group A, member 3
9315	NREP	UP				X				neuronal regeneration related protein
4928	NUP98	DOWN							X	nucleoporin 98kDa
8572	PDLIM4	UP					X			PDZ and LIM domain 4
5371	PML	DOWN							X	promyelocytic leukemia
7799	PRDM2	DOWN							X	PR domain containing 2, with ZNF domain
5567	PRKACB	UP					X			protein kinase, cAMP-dependent, catalytic, beta
5728	PTEN	DOWN		X	X				X	phosphatase and tensin homolog

9401	RECQL4	DOWN							X	RecQ protein-like 4
5970	RELA	DOWN		X					X	v-rel reticuloendotheliosis viral oncogene homolog A (avian)
388	RHOB	DOWN							X	ras homolog family member B
22800	RRAS2	UP				X				related RAS viral (r-ras) oncogene homolog 2
23623	RUSC1	UP					X			RUN and SH3 domain containing 1
6284	S100A13	UP							X	S100 calcium binding protein A13
6414	SEPP1	UP			X					selenoprotein P, plasma, 1
946	SIGLEC6	UP			X					sialic acid binding Ig-like lectin 6
6886	TAL1	DOWN							X	T-cell acute lymphocytic leukemia 1
7037	TFRC	UP		X	X				X	transferrin receptor (p90, CD71)
7040	TGFB1	UP			X					transforming growth factor, beta 1
7043	TGFB3	DOWN			X					transforming growth factor, beta 3
7140	TNNT3	DOWN			X					troponin T type 3 (skeletal, fast)
9099	USP2	UP	X							ubiquitin specific peptidase 2
7428	VHL	UP		X					X	von Hippel-Lindau tumor suppressor, E3 ubiquitin protein ligase
	Total genes:	93								
	List A total:	539								

**List B**			Cancer-related genes:							
			List 1	List 2	List 3	List 4	List 5	List 6	List 7	
GeneID	Gene name	FSHD	6	4	10	10	6	9	16	Gene description
231	AKR1B1	DOWN			X					aldo-keto reductase family 1, member B1 (aldose reductase)
10000	AKT3	DOWN							X	v-akt murine thymoma viral oncogene homolog 3 (protein kinase B, gamma)
214	ALCAM	DOWN					X			activated leukocyte cell adhesion molecule
4329	ALDH6A1	DOWN	X							aldehyde dehydrogenase 6 family, member A1
367	AR	DOWN			X				X	androgen receptor
604	BCL6	DOWN							X	B-cell CLL/lymphoma 6
800	CALD1	DOWN					X			caldesmon 1
868	CBLB	DOWN							X	Cbl proto-oncogene, E3 ubiquitin protein ligase B
1012	CDH13	DOWN			X					cadherin 13, H-cadherin (heart)
1056	CEL	DOWN						X		carboxyl ester lipase (bile salt-stimulated lipase)
1063	CENPF	DOWN					X		X	centromere protein F, 350/400kDa
3075	CFH	DOWN			X					complement factor H
1653	DDX1	DOWN				X				DEAD (Asp-Glu-Ala-Asp) box helicase 1
2202	EFEMP1	UP			X					EGF containing fibulin-like extracellular matrix protein 1
2191	FAP	DOWN						X		fibroblast activation protein, alpha
2271	FH	DOWN		X					X	fumarate hydratase
10468	FST	DOWN				X				follistatin
2521	FUS	DOWN		X					X	fused in sarcoma
2535	FZD2	DOWN				X				frizzled family receptor 2
11146	GLMN	DOWN						X	X	glomulin, FKBP associated protein
29889	GNL2	DOWN	X			X				guanine nucleotide binding protein-like 2 (nucleolar)
3065	HDAC1	DOWN						X		histone deacetylase 1
3066	HDAC2	DOWN				X				histone deacetylase 2
3092	HIP1	DOWN							X	huntingtin interacting protein 1
8364	HIST1H4C	DOWN				X				histone cluster 1, H4c
3181	HNRNPA2B1	DOWN						X		heterogeneous nuclear ribonucleoprotein A2/B1
3329	HSPD1	DOWN						X	X	heat shock 60kDa protein 1 (chaperonin)
3609	ILF3	DOWN	X							interleukin enhancer binding factor 3, 90kDa
3667	IRS1	DOWN			X					insulin receptor substrate 1
5927	KDM5A	DOWN							X	lysine (K)-specific demethylase 5A
9933	KIAA0020	DOWN				X				KIAA0020
4437	MSH3	DOWN							X	mutS homolog 3 (E. coli)
2956	MSH6	DOWN			X	X			X	mutS homolog 6 (E. coli)
10797	MTHFD2	DOWN				X		X		methylenetetrahydrofolate dehydrogenase (NADP+ dependent) 2, methenyltetrahydrofolate cyclohydrolase
4610	MYCL1	DOWN			X					v-myc myelocytomatosis viral oncogene homolog 1, lung carcinoma derived (avian)
9315	NREP	DOWN				X				neuronal regeneration related protein
142	PARP1	DOWN		X						poly (ADP-ribose) polymerase 1
27250	PDCD4	DOWN							X	programmed cell death 4 (neoplastic transformation inhibitor)
56937	PMEPA1	DOWN	X							prostate transmembrane protein, androgen induced 1
5587	PRKD1	DOWN			X					protein kinase D1
5696	PSMB8	DOWN	X							proteasome (prosome, macropain) subunit, beta type, 8 (large multifunctional peptidase 7)
861	RUNX1	DOWN		X					X	runt-related transcription factor 1
6275	S100A4	DOWN					X			S100 calcium binding protein A4
6597	SMARCA4	DOWN						X		SWI/SNF related, matrix associated, actin dependent regulator of chromatin, subfamily a, member 4
6427	SRSF2	DOWN					X			serine/arginine-rich splicing factor 2
6745	SSR1	DOWN						X		signal sequence receptor, alpha
6890	TAP1	DOWN	X							transporter 1, ATP-binding cassette, sub-family B (MDR/TAP)
6925	TCF4	DOWN					X			transcription factor 4
7078	TIMP3	DOWN			X					TIMP metallopeptidase inhibitor 3
7175	TPR	DOWN							X	translocated promoter region, nuclear basket protein
7412	VCAM1	DOWN			X					vascular cell adhesion molecule 1
	Total genes:	51								
	List B total:	296								

**List Ca**			Cancer-related genes:							
			List 1	List 2	List 3	List 4	List 5	List 6	List 7	
GeneID	Gene name	FSHD	1	0	14	0	1	0	10	Gene description
58	ACTA1	UP			X					actin, alpha 1, skeletal muscle
100	ADA	DOWN			X					adenosine deaminase
217	ALDH2	UP							X	aldehyde dehydrogenase 2 family (mitochondrial)
241	ALOX5AP	DOWN							X	arachidonate 5-lipoxygenase-activating protein
637	BID	DOWN	X							BH3 interacting domain death agonist
805	CALM2	UP			X					calmodulin 2 (phosphorylase kinase, delta)
10983	CCNI	DOWN			X					cyclin I
3075	CFH	DOWN			X					complement factor H
1345	COX6C	DOWN							X	cytochrome c oxidase subunit VIc
1508	CTSB	UP							X	cathepsin B
7913	DEK	UP							X	DEK oncogene
1674	DES	UP			X					desmin
2273	FHL1	DOWN			X					four and a half LIM domains 1
2597	GAPDH	UP			X					glyceraldehyde-3-phosphate dehydrogenase
27259	HPLH1	DOWN			X					hemophagocytic lymphohistiocytosis 1
11343	MGLL	DOWN			X					monoglyceride lipase
4946	OAZ1	UP			X					ornithine decarboxylase antizyme 1
5052	PRDX1	DOWN							X	peroxiredoxin 1
5684	PSMA3	UP			X					proteasome (prosome, macropain) subunit, alpha type, 3
5743	PTGS2	UP							X	prostaglandin-endoperoxide synthase 2 (prostaglandin G/H synthase and cyclooxygenase)
389	RHOC	UP							X	ras homolog family member C
11236	RNF139	UP							X	ring finger protein 139
57142	RTN4	DOWN							X	reticulon 4
7135	TNNI1	UP			X					troponin I type 1 (skeletal, slow)
7136	TNNI2	UP			X					troponin I type 2 (skeletal, fast)
7140	TNNT3	UP			X					troponin T type 3 (skeletal, fast)
7169	TPM2	UP					X			tropomyosin 2 (beta)
	Total genes:	27								
	List Ca total:	207								

List Cb			Cancer-related genes:							
			List 1	List 2	List 3	List 4	List 5	List 6	List 7	
GeneID	Gene name	FSHD	0	0	13	0	2	0	9	Gene description
58	ACTA1	UP			X					actin, alpha 1, skeletal muscle
100	ADA	UP			X					adenosine deaminase
217	ALDH2	DOWN							X	aldehyde dehydrogenase 2 family (mitochondrial)
573	BAG1	UP					X			BCL2-associated athanogene
805	CALM2	UP			X					calmodulin 2 (phosphorylase kinase, delta)
10983	CCNI	DOWN			X					cyclin I
3075	CFH	UP			X					complement factor H
1345	COX6C	UP							X	cytochrome c oxidase subunit VIc
1508	CTSB	UP							X	cathepsin B
7913	DEK	UP							X	DEK oncogene
1674	DES	DOWN			X					desmin
2273	FHL1	UP			X					four and a half LIM domains 1
2597	GAPDH	UP			X					glyceraldehyde-3-phosphate dehydrogenase
27259	HPLH1	UP			X					hemophagocytic lymphohistiocytosis 1
4946	OAZ1	UP			X					ornithine decarboxylase antizyme 1
5052	PRDX1	UP							X	peroxiredoxin 1
5684	PSMA3	UP			X					proteasome (prosome, macropain) subunit, alpha type, 3
5743	PTGS2	UP							X	prostaglandin-endoperoxide synthase 2 (prostaglandin G/H synthase and cyclooxygenase)
389	RHOC	DOWN							X	ras homolog family member C
11236	RNF139	UP							X	ring finger protein 139
57142	RTN4	UP							X	reticulon 4
7135	TNNI1	DOWN			X					troponin I type 1 (skeletal, slow)
7136	TNNI2	UP			X					troponin I type 2 (skeletal, fast)
7140	TNNT3	DOWN			X					troponin T type 3 (skeletal, fast)
7169	TPM2	DOWN					X			tropomyosin 2 (beta)
	Total genes:	25								
	List Cb total:	177								

**List Cc**			Cancer-related genes:							
			List 1	List 2	List 3	List 4	List 5	List 6	List 7	
GeneID	Gene name	FSHD	0	0	13	0	2	0	9	Gene description
58	ACTA1	UP			X					actin, alpha 1, skeletal muscle
100	ADA	UP			X					adenosine deaminase
217	ALDH2	DOWN							X	aldehyde dehydrogenase 2 family (mitochondrial)
573	BAG1	UP					X			BCL2-associated athanogene
805	CALM2	UP			X					calmodulin 2 (phosphorylase kinase, delta)
10983	CCNI	DOWN			X					cyclin I
3075	CFH	DOWN			X					complement factor H
1345	COX6C	DOWN							X	cytochrome c oxidase subunit VIc
1508	CTSB	UP							X	cathepsin B
7913	DEK	DOWN							X	DEK oncogene
1674	DES	UP			X					desmin
2273	FHL1	DOWN			X					four and a half LIM domains 1
2597	GAPDH	UP			X					glyceraldehyde-3-phosphate dehydrogenase
27259	HPLH1	DOWN			X					hemophagocytic lymphohistiocytosis 1
4946	OAZ1	UP			X					ornithine decarboxylase antizyme 1
5052	PRDX1	UP							X	peroxiredoxin 1
5684	PSMA3	DOWN			X					proteasome (prosome, macropain) subunit, alpha type, 3
5743	PTGS2	UP							X	prostaglandin-endoperoxide synthase 2 (prostaglandin G/H synthase and cyclooxygenase)
389	RHOC	UP							X	ras homolog family member C
11236	RNF139	UP							X	ring finger protein 139
57142	RTN4	DOWN							X	reticulon 4
7135	TNNI1	UP			X					troponin I type 1 (skeletal, slow)
7136	TNNI2	DOWN			X					troponin I type 2 (skeletal, fast)
7140	TNNT3	DOWN			X					troponin T type 3 (skeletal, fast)
7169	TPM2	UP					X			tropomyosin 2 (beta)
	Total genes:	25								
	List Cc total:	177								

**List D**			Cancer-related genes:							
			List 1	List 2	List 3	List 4	List 5	List 6	List 7	
GeneID	Gene name	FSHD	1	0	0	0	0	0	3	Gene description
1620	DBC1	UP				X				deleted in bladder cancer 1
23266	LPHN2	UP							X	latrophilin 2
5918	RARRES1	UP							X	retinoic acid receptor responder (tazarotene induced) 1
7058	THBS2	UP							X	thrombospondin 2
8840	WISP1	UP	X							WNT1 inducible signaling pathway protein 1
	Total genes:	5								
	List D total:	32								

**List Ea**			Cancer-related genes:							
			List 1	List 2	List 3	List 4	List 5	List 6	List 7	
GeneID	Gene name	FSHD	6	1	13	5	8	0	15	Gene description
59	ACTA2	UP			X					actin, alpha 2, smooth muscle, aorta
70	ACTC1	UP			X					actin, alpha, cardiac muscle 1
301	ANXA1	UP			X					annexin A1
8853	ASAP2	UP			X					ArfGAP with SH3 domain, ankyrin repeat and PH domain 2
580	BARD1	DOWN							X	BRCA1 associated RING domain 1
800	CALD1	UP					X			caldesmon 1
960	CD44	UP			X					CD44 molecule (Indian blood group)
3075	CFH	UP			X					complement factor H
1191	CLU	UP	X							clusterin
23406	COTL1	UP					X			coactosin-like 1 (Dictyostelium)
1490	CTGF	UP							X	connective tissue growth factor
11034	DSTN	UP					X			destrin (actin depolymerizing factor)
1871	E2F3	UP	X						X	E2F transcription factor 3
2192	FBLN1	UP					X			fibulin 1
2329	FMO2	DOWN	X							flavin containing monooxygenase 2 (non-functional)
84624	FNDC1	UP			X					fibronectin type III domain containing 1
3090	HIC1	UP							X	hypermethylated in cancer 1
3383	ICAM1	UP			X					intercellular adhesion molecule 1
23463	ICMT	UP				X				isoprenylcysteine carboxyl methyltransferase
3643	INSR	DOWN			X					insulin receptor
3667	IRS1	DOWN			X					insulin receptor substrate 1
3727	JUND	UP							X	jun D proto-oncogene
23313	KIAA0930	UP					X			KIAA0930
378938	MALAT1	DOWN				X				metastasis associated lung adenocarcinoma transcript 1 (non-protein coding)
4233	MET	DOWN		X	X				X	met proto-oncogene (hepatocyte growth factor receptor)
8731	MET	DOWN			X					met proto-oncogene (hepatocyte growth factor receptor)
10962	MLLT11	UP					X		X	myeloid/lymphoid or mixed-lineage leukemia (trithorax homolog, Drosophila); translocated to, 11
4478	MSN	UP							X	moesin
9961	MVP	UP							X	major vault protein
3164	NR4A1	UP			X					nuclear receptor subfamily 4, group A, member 1
9315	NREP	DOWN				X				neuronal regeneration related protein
4915	NTRK2	UP							X	neurotrophic tyrosine kinase, receptor, type 2
8572	PDLIM4	UP					X			PDZ and LIM domain 4
5788	PTPRC	UP				X				protein tyrosine phosphatase, receptor type, C
80031	SEMA6D	DOWN	X							sema domain, transmembrane domain (TM), and cytoplasmic domain, (semaphorin) 6D
6418	SET	DOWN							X	SET nuclear oncogene
23235	SIK2	UP	X							salt-inducible kinase 2
8148	TAF15	DOWN							X	TAF15 RNA polymerase II, TATA box binding protein (TBP)-associated factor, 68kDa
7057	THBS1	UP					X		X	thrombospondin 1
7058	THBS2	UP							X	thrombospondin 2
10376	TUBA1B	UP	X							tubulin, alpha 1b
7431	VIM	UP			X					vimentin
26137	ZBTB20	DOWN				X				zinc finger and BTB domain containing 20
677	ZFP36L1	UP							X	ZFP36 ring finger protein-like 1
	Total genes:	44								
	List Ea total:	326								

**List Eb**			Cancer-related genes:							
			List 1	List 2	List 3	List 4	List 5	List 6	List 7	
GeneID	Gene name	FSHD	1	1	5	3	3	0	7	Gene description
580	BARD1	DOWN							X	BRCA1 associated RING domain 1
960	CD44	UP			X					CD44 molecule (Indian blood group)
23406	COTL1	UP					X			coactosin-like 1 (Dictyostelium)
1490	CTGF	UP							X	connective tissue growth factor
3090	HIC1	UP							X	hypermethylated in cancer 1
3383	ICAM1	UP			X					intercellular adhesion molecule 1
23463	ICMT	UP				X				isoprenylcysteine carboxyl methyltransferase
3643	INSR	DOWN			X					insulin receptor
3727	JUND	UP							X	jun D proto-oncogene
23313	KIAA0930	UP					X			KIAA0930
4233	MET	DOWN		X	X				X	met proto-oncogene (hepatocyte growth factor receptor)
8731	MET	DOWN			X					met proto-oncogene (hepatocyte growth factor receptor)
9315	NREP	DOWN				X				neuronal regeneration related protein
80031	SEMA6D	DOWN	X							sema domain, transmembrane domain (TM), and cytoplasmic domain, (semaphorin) 6D
6418	SET	DOWN							X	SET nuclear oncogene
7057	THBS1	UP					X		X	thrombospondin 1
26137	ZBTB20	DOWN				X				zinc finger and BTB domain containing 20
677	ZFP36L1	UP							X	ZFP36 ring finger protein-like 1
	Total genes:	18								
	List Eb total:	156								

List Fa			Cancer-related genes:							
			List 1	List 2	List 3	List 4	List 5	List 6	List 7	
GeneID	Gene name	FSHD	14	3	11	24	5	9	23	Gene description
231	AKR1B1	UP			X					aldo-keto reductase family 1, member B1 (aldose reductase)
80210	ARMC9	UP				X				armadillo repeat containing 9
6790	AURKA	DOWN	X							aurora kinase A
79870	BAALC	UP							X	brain and acute leukemia, cytoplasmic
641	BLM	DOWN							X	Bloom syndrome, RecQ helicase-like
672	BRCA1	DOWN		X	X				X	breast cancer 1, early onset
675	BRCA2	DOWN			X					breast cancer 2, early onset
2187	BRCA2	DOWN				X				breast cancer 2, early onset
701	BUB1B	DOWN				X			X	BUB1 mitotic checkpoint serine/threonine kinase B
10826	C5orf4	UP	X							chromosome 5 open reading frame 4
57082	CASC5	DOWN	X						X	cancer susceptibility candidate 5
890	CCNA2	DOWN				X				cyclin A2
993	CDC25A	DOWN				X			X	cell division cycle 25A
990	CDC6	DOWN							X	cell division cycle 6
983	CDK1	DOWN						X		cyclin-dependent kinase 1
1017	CDK2	DOWN			X					cyclin-dependent kinase 2
1062	CENPE	DOWN				X				centromere protein E, 312kDa
1063	CENPF	DOWN					X		X	centromere protein F, 350/400kDa
2491	CENPI	DOWN				X				centromere protein I
55165	CEP55	DOWN	X							centrosomal protein 55kDa
79077	DCTPP1	DOWN	X							dCTP pyrophosphatase 1
81624	DIAPH3	DOWN				X				diaphanous homolog 3 (Drosophila)
9787	DLGAP5	DOWN				X				discs, large (Drosophila) homolog-associated protein 5
1786	DNMT1	DOWN	X						X	DNA (cytosine-5-)-methyltransferase 1
2202	EFEMP1	DOWN			X					EGF containing fibulin-like extracellular matrix protein 1
2175	FANCA	DOWN							X	Fanconi anemia, complementation group A
2187	FANCB	DOWN				X				Fanconi anemia, complementation group B
2176	FANCC	DOWN							X	Fanconi anemia, complementation group C
2192	FBLN1	UP					X			fibulin 1
2237	FEN1	DOWN	X							flap structure-specific endonuclease 1
2272	FHIT	UP							X	fragile histidine triad
23767	FLRT3	UP			X					fibronectin leucine rich transmembrane protein 3
2305	FOXM1	DOWN							X	forkhead box M1
10468	FST	DOWN				X				follistatin
2495	FTH1	UP			X					ferritin, heavy polypeptide 1
8364	HIST1H4C	DOWN				X				histone cluster 1, H4c
55355	HJURP	DOWN	X							Holliday junction recognition protein
3207	HOXA11	UP		X					X	homeobox A11
3229	HOXC13	UP		X						homeobox C13
128239	IQGAP3	DOWN	X							IQ motif containing GTPase activating protein 3
3832	KIF11	DOWN				X				kinesin family member 11
9928	KIF14	DOWN							X	kinesin family member 14
9493	KIF23	DOWN				X				kinesin family member 23
24137	KIF4A	DOWN					X			kinesin family member 4A
55388	MCM10	DOWN				X				minichromosome maintenance complex component 10
4172	MCM3	DOWN						X		minichromosome maintenance complex component 3
4173	MCM4	DOWN				X				minichromosome maintenance complex component 4
4175	MCM6	DOWN				X				minichromosome maintenance complex component 6
4288	MKI67	DOWN			X					antigen identified by monoclonal antibody Ki-67
4311	MME	UP							X	membrane metallo-endopeptidase
4436	MSH2	DOWN		X	X				X	mutS homolog 2, colon cancer, nonpolyposis type 1 (E. coli)
10797	MTHFD2	DOWN				X		X		methylenetetrahydrofolate dehydrogenase (NADP+ dependent) 2, methenyltetrahydrofolate cyclohydrolase
4582	MUC1	UP			X					mucin 1, cell surface associated
64151	NCAPG	DOWN					X			non-SMC condensin I complex, subunit G
10403	NDC80	DOWN				X				NDC80 kinetochore complex component
8828	NRP2	UP	X							neuropilin 2
23165	NUP205	DOWN						X		nucleoporin 205kDa
4998	ORC1	DOWN				X				origin recognition complex, subunit 1
5074	PAWR	DOWN							X	PRKC, apoptosis, WT1, regulator
5077	PAX3	UP							X	paired box 3
5347	PLK1	DOWN						X	X	polo-like kinase 1
5424	POLD1	DOWN						X		polymerase (DNA directed), delta 1, catalytic subunit
5427	POLE2	DOWN				X				polymerase (DNA directed), epsilon 2, accessory subunit
9055	PRC1	DOWN	X							protein regulator of cytokinesis 1
5557	PRIM1	DOWN				X				primase, DNA, polypeptide 1 (49kDa)
5793	PTPRG	UP							X	protein tyrosine phosphatase, receptor type, G
29127	RACGAP1	DOWN					X			Rac GTPase activating protein 1
10635	RAD51AP1	DOWN			X					RAD51 associated protein 1
5984	RFC4	DOWN						X		replication factor C (activator 1) 4, 37kDa
54908	SPDL1	DOWN				X				spindle apparatus coiled-coil protein 1
7078	TIMP3	DOWN			X					TIMP metallopeptidase inhibitor 3
10673	TNFSF13B	DOWN	X							tumor necrosis factor (ligand) superfamily, member 13b
7153	TOP2A	DOWN						X		topoisomerase (DNA) II alpha 170kDa
22974	TPX2	DOWN						X		TPX2, microtubule-associated, homolog (Xenopus laevis)
7272	TTK	DOWN	X							TTK protein kinase

55055	ZWILCH	DOWN	X							zwilch kinetochore protein
	Total genes:	78								
	List Fa total:	395								

**List Fb**			Cancer-related genes:							
			List 1	List 2	List 3	List 4	List 5	List 6	List 7	
GeneID	Gene name	FSHD	2	0	4	2	1	1	3	Gene description
92	ACVR2A	DOWN							X	activin A receptor, type IIA
231	AKR1B1	UP			X					aldo-keto reductase family 1, member B1 (aldose reductase)
79870	BAALC	UP							X	brain and acute leukemia, cytoplasmic
595	CCND1	UP			X				X	cyclin D1
2202	EFEMP1	DOWN			X					EGF containing fibulin-like extracellular matrix protein 1
23767	FLRT3	UP			X					fibronectin leucine rich transmembrane protein 3
3037	HAS2	UP				X				hyaluronan synthase 2
56243	KIAA1217	UP	X							KIAA1217
4311	MME	UP							X	membrane metallo-endopeptidase
10797	MTHFD2	DOWN				X		X		methylenetetrahydrofolate dehydrogenase (NADP+ dependent) 2, methenyltetrahydrofolate cyclohydrolase
4628	MYH10	DOWN					X			myosin, heavy chain 10, non-muscle
8470	SORBS2	DOWN	X							sorbin and SH3 domain containing 2
	Total genes:	12								
	List Fb total:	111								

**List Ga**			Cancer-related genes:							
			List 1	List 2	List 3	List 4	List 5	List 6	List 7	
GeneID	Gene name	FSHD	2	0	12	1	1	0	11	Gene description
59	ACTA2	UP			X					actin, alpha 2, smooth muscle, aorta
70	ACTC1	UP			X					actin, alpha, cardiac muscle 1
92	ACVR2A	DOWN							X	activin A receptor, type IIA
241	ALOX5AP	UP							X	arachidonate 5-lipoxygenase-activating protein
10203	CALCRL	UP			X					calcitonin receptor-like
960	CD44	UP			X					CD44 molecule (Indian blood group)
963	CD53	UP				X			X	CD53 molecule
3075	CFH	UP			X					complement factor H
7123	CLEC3B	UP	X							C-type lectin domain family 3, member B
1277	COL1A1	UP							X	collagen, type I, alpha 1
2202	EFEMP1	UP			X					EGF containing fibulin-like extracellular matrix protein 1
2178	FANCE	DOWN							X	Fanconi anemia, complementation group E
2353	FOS	UP			X					FBJ murine osteosarcoma viral oncogene homolog
166647	GPR125	DOWN					X			G protein-coupled receptor 125
3479	IGF1	UP			X					insulin-like growth factor 1 (somatomedin C)
22807	IKZF2	DOWN							X	IKAROS family zinc finger 2 (Helios)
3690	ITGB3	UP			X					integrin, beta 3 (platelet glycoprotein IIIa, antigen CD61)
3702	ITK	UP							X	IL2-inducible T-cell kinase
9211	LGI1	DOWN							X	leucine-rich, glioma inactivated 1
3164	NR4A1	UP			X					nuclear receptor subfamily 4, group A, member 1
8643	PTCH2	UP							X	patched 2
5968	REG1B	UP			X					regenerating islet-derived 1 beta
80031	SEMA6D	DOWN	X							sema domain, transmembrane domain (TM), and cytoplasmic domain, (semaphorin) 6D
5552	SRGN	UP			X					serglycin
3371	TNC	UP							X	tenascin C
7139	TNNT2	UP			X					troponin T type 2 (cardiac)
7170	TPM3	DOWN							X	tropomyosin 3
	Total genes:	27								
	List Ga total:	272								

**List Gb**			Cancer-related genes:							
			List 1	List 2	List 3	List 4	List 5	List 6	List 7	
GeneID	Gene name	FSHD	2	0	7	1	1	0	9	Gene description
59	ACTA2	UP			X					actin, alpha 2, smooth muscle, aorta
70	ACTC1	UP			X					actin, alpha, cardiac muscle 1
92	ACVR2A	DOWN							X	activin A receptor, type IIA
241	ALOX5AP	UP							X	arachidonate 5-lipoxygenase-activating protein
963	CD53	UP				X			X	CD53 molecule
7123	CLEC3B	UP	X							C-type lectin domain family 3, member B
2202	EFEMP1	UP			X					EGF containing fibulin-like extracellular matrix protein 1
2353	FOS	UP			X					FBJ murine osteosarcoma viral oncogene homolog
166647	GPR125	DOWN					X			G protein-coupled receptor 125
22807	IKZF2	DOWN							X	IKAROS family zinc finger 2 (Helios)
3702	ITK	UP							X	IL2-inducible T-cell kinase
9211	LGI1	DOWN							X	leucine-rich, glioma inactivated 1
3164	NR4A1	UP			X					nuclear receptor subfamily 4, group A, member 1
8643	PTCH2	UP							X	patched 2
5968	REG1B	UP			X					regenerating islet-derived 1 beta
80031	SEMA6D	DOWN	X							sema domain, transmembrane domain (TM), and cytoplasmic domain, (semaphorin) 6D
5552	SRGN	UP			X					serglycin
3371	TNC	UP							X	tenascin C
7139	TNNT2	UP			X					troponin T type 2 (cardiac)
7170	TPM3	DOWN							X	tropomyosin 3
	Total genes:	20								
	List Gb total:	162								
