# Supplementary material for: Cancer-related genes in the transcription signature of facioscapulohumeral dystrophy myoblasts and myotubes
Source: J Cell Mol Med. 2013 Dec 17;18(2):208–17. doi: 10.1111/jcmm.12182 (PMC3930408; doi:10.1111/jcmm.12182)
Supplement: Supplementary file 6 [file jcmm0018-0208-sd6.docx]

**Table S6.** Samples used in the analysis. MT - myotubes MB - myoblasts.

| Sample | Type | Patient | Tissue | Age | Sex | D4Z4 repeats |
| --- | --- | --- | --- | --- | --- | --- |
| Normal | | | | | | |
| 1 | MB, MT | NO45 | quadriceps | 35 | F | ND |
| 2 | MB, MT | NO40 | quadriceps | 46 | M | ND |
| 3 | MB, MT | NO42 | quadriceps | 24 | F | ND |
| 4 | MB, MT | NO44 | quadriceps | 29 | M | ND |
| 5 | MB, MT | NO47 | quadriceps | 43 | M | ND |
| FSHD | | | | | | |
| 1 | MB, MT | FSHD10 | trapezius | 31 | M | 5 |
| 2 | MB, MT | MO41 | quadriceps | 23 | F | 8 |
| 3 | MB, MT | MO44 | pyramidal | 54 | F | 5/7 |
| 4 | MB, MT | MO47 | quadriceps | 38 | F | 7 |
| 5 | MB, MT | MO54 | quadriceps | 25 | M | 4 |
